# Longitudinal functional changes with clinically significant radiographic progression in idiopathic pulmonary fibrosis: are we following the right parameters?

**DOI:** 10.1186/s12931-020-01371-7

**Published:** 2020-05-19

**Authors:** Nada Taha, Dejanira D’Amato, Karishma Hosein, Tiziana Ranalli, Gianluigi Sergiacomi, Maurizio Zompatori, Marco Mura

**Affiliations:** 1 Division of Respirology, Western University, London, Ontario Canada; 2 Diagnostica per Immagini e Radiologia Interventistica, Policlinico Tor Vergata, University of Rome “Tor Vergata”, Rome, Italy; 3 Radiologia, MultiMedica Group, I.R.C.C.S. San Giuseppe Hospital, Milan, Italy; 4 London Health Science Centre, Victoria Hospital, 800 Commissioners Road East Room E6-203, London, Ontario N6A 5W9 Canada

**Keywords:** Idiopathic pulmonary fibrosis, High resolution chest CT scan, Lung function, Fibrosis score, Survival

## Abstract

**Background:**

Progression of the disease in idiopathic pulmonary fibrosis (IPF) is difficult to predict, due to its variable and heterogenous course. The relationship between radiographic progression and functional decline in IPF is unclear. We sought to confirm that a simple HRCT fibrosis visual score is a reliable predictor of mortality in IPF, when longitudinally followed; and to ascertain which pulmonary functional variables best reflect clinically significant radiographic progression.

**Methods:**

One-hundred-twenty-three consecutive patients with IPF from 2 centers were followed for an average of 3 years. Longitudinal changes of HRCT fibrosis scores, forced vital capacity (FVC), total lung capacity and diffusing lung capacity for carbon monoxide were considered. HRCTs were scored by 2 chest radiologists. The primary outcome was lung transplant (LTx)-free survival after the follow-up HRCT.

**Results:**

During the follow-up period, 43 deaths and 11 LTx occurred. On average, the HRCT fibrosis score increased significantly, and a longitudinal increase > 7% predicted LTx-free survival significantly, with good specificity, but limited sensitivity. The correlation between radiographic and functional progression was moderately significant. HRCT progression and FVC decline predicted LTx-free survival independently and significantly, with better sensitivity, but worse specificity for a ≥ 5% decline of FVC. However, the area under the curve towards LTx-survival were only 0.61 and 0.62, respectively.

**Conclusions:**

The HRCT fibrosis visual score is a reliable and responsive tool to detect clinically meaningful disease progression. Although no individual pulmonary function test closely reflects radiographic progression, a longitudinal FVC decline improves sensitivity in the detection of clinically significant disease progression. However, the accuracy of these methods remains limited, and better prognostication models need to be found.

## Background

One of the major challenges in the management of idiopathic pulmonary fibrosis (IPF) is predicting the clinical course of the disease in an individual patient, given its variability and heterogeneity [1]. The detection of clinically significant progression is paramount to take decisions in terms of prognostication, anti-fibrotic therapy, lung transplant (LTx) assessment and listing. The use of clinically meaningful endpoints is also decisive in the conduction of new clinical trials in IPF [2].

Progression of disease IPF usually occurs with gradual worsening of radiographic fibrotic changes and physiological studies, including pulmonary function tests (PFTs) [3] and 6-min walk test [4], in addition to clinical symptoms, including dyspnea and cough. Consequently, commonly used predictors of disease progression include forced vital capacity (FVC) decline [5] and the repeat use of high-resolution chest CT scan (HRCT) [6]. The decline in the FVC has been in fact the driving parameter for the approval of anti-fibrotic drugs in IPF [7, 8]. This has been preferred over the use of HRCT as an endpoint, given the concerns over exposure to radiation and cost.

Several methods exist to evaluate the extent of lung fibrosis on HRCT and to assess disease progression. These include visual scores [6, 9] and automated methods [10–13]. One recognized limit in the use of HRCT in interstitial lung disease is interobserver variability in terms of pattern recognition [14, 15]. Despite the fact that automated methods of fibrosis scoring on HRCT may not be readily available in the majority of institutions, only a surprisingly small number of studies investigated the use of HRCT fibrosis visual scores in the longitudinal assessment of IPF [16, 17]. At the same time, concerns have arisen on the reliability of FVC alone in determining therapeutic decisions. These include missing progression in patients with stable FVC [18], observed intra-subject variability [19], and the fact that FVC decline itself is slowed down by anti-fibrotic therapy [2]. Other factors that can contribute to lung function fluctuations are represented by temporal variability of FVC during acute exacerbations [20] and by lack of compliance to prescribed therapy.

Previous studies investigated the relationship between concomitant radiographic and functional progression in IPF, but they were limited by either short follow-up period [6, 21–23], and/or by the inclusion of a small cohort [22–24]. Moreover, none of the previous studies applied c-statistics to investigate the accuracy of radiographic vs. functional progression to predict outcome. Collinearity between functional and radiographic progression was not studied either.

We previously demonstrated that baseline HRCT fibrosis visual score predicted 3-year survival in a cohort of patients newly diagnosed with IPF significantly [25]. It remains to be established whether longitudinal changes of an HRCT visual fibrosis score represent a reliable predictor of progression in IPF. Furthermore, the relationship between radiographic and functional progression in IPF remains to be ascertained.

The objectives of this multicenter study were therefore: 1) to confirm that a simple HRCT fibrosis visual score is a reliable predictor of mortality in IPF, when longitudinally followed; 2) to ascertain which pulmonary functional variables best reflect clinically significant progression of disease seen on HRCT; 3) to assess the accuracy of mortality prediction of both radiographic and functional progression.

## Methods

### Subjects

Consecutive patients diagnosed with IPF were enrolled in local databases and followed by 2 centres, London, ON, Canada and Rome, Italy. The diagnosis of IPF was based on the ATS/ERS/JRS/ALAT guidelines [26] in the context of multi-disciplinary discussion (MDD) [27]. Longitudinal data were retrospectively collected. Patients who had 2 consecutive HRCT and corresponding PFT within 3 months from the HRCT scan date, and at least 1 year of clinical follow up from the time of diagnosis were included in the study. Patients with any other type of interstitial lung disease were excluded.

The follow-up HRCT was ordered by the attending physician as clinically indicated. HRCTs are routinely ordered in our practices every 2 years from the time of diagnosis, and whenever progression of disease or acute exacerbations are suspected. Variables considered at baseline and follow-up assessment included an HRCT fibrosis visual score and measures of lung function, including forced vital capacity (FVC), total lung capacity (TLC), and diffusing lung capacity for carbon monoxide (DLCO). Twenty-nine patients were not able to perform a DLCO either at baseline or at follow-up assessment. Nineteen patients were not able to perform a TLC either at baseline or at follow-up assessment. There were no other missing data. Pulmonary function tests were performed according to ERS/ATS guidelines [28–30].

The primary outcome of the study was lung transplant-free survival from the date of the follow-up HRCT. The study was approved by the research ethics boards of Western University (protocol n. 101386) and University of Rome “Tor Vergata” (protocol n.175/15).

### HRCT

The overall extent of pulmonary fibrosis was evaluated with a quantitative visual score [9, 25]. Two chest radiologists from different institutions, with specific interstitial lung disease expertise and > 20 years experience (G.S., M.Z.), who were blinded to clinical information, separately assessed the pattern and scored each case. Interobserver variability was assessed, and the average score was calculated. HRCT images were assessed for the presence and extent of parenchymal abnormalities, including reticular opacities, ground-glass opacities, traction bronchiectasis and honeycombing. A 5-point scale (0 = absence of lesions, 1, 2, 3, 4 = extent of lesions, respectively, < 25, 25–50, 50–75, > 75%) was used to determine the extent of overall fibrotic lung disease. The scores assigned for each scan at 4 predefined levels (aortic arch, bronchus intermedius, pulmonary veins, lowest scan) and each hemithorax were summed, and a final value was obtained: score = 100/maximum predicted value (equivalent to 8 times the number of scans performed). The extent of fibrosis was expressed as a percentage of the total lung volume. Baseline and follow-up HRCT fibrosis visual scores were calculated.

### Statistical analysis

Values are expressed as mean ± standard deviation. HRCT fibrosis score intervals were determined as: 0–10% -1, 11–20% -2, 21–30% - 3, and here on. Weighted kappa coefficient was then used to calculate interobserver agreement in quantitative fibrosis scoring [31]. The Kolmogorov-Smirnov test was used for distribution analysis. Spearman coefficients were then used to assess linear association between variables. We calculated the variance inflation factors of the predicting variables to rule out the possibility of multicollinearity and demonstrate that the variables were truly independent [32]. Receiver operating characteristic (ROC) analysis (c-statistics) was used to determine best cut points of each variable towards the endpoints, by examining accuracy (sum of sensitivity and specificity) of predicting endpoints. Univariate and multivariate Cox proportional hazards regression analyses were performed identifying the significance of variables predicting the endpoint. *p* values < 0.05 were regarded as significant. GraphPad (MacKiev, San Diego, CA), JMP (SAS Institute, Cary, NC), MedCalc (MedCalc, Mariakerke, Belgium) and Stata (Stata Corp, College Station, TX) softwares were used.

## Results

One hundred twenty-three patients from the 2 centers were included in the study. Demographic, clinical and functional characteristics are shown in Table 1. Eighty-six patients (70%) were diagnosed with based on clinical-radiographic criteria, and thirty-seven (30%) based on clinical-radiographic-pathologic criteria. The average interval between the baseline and follow-up HRCTs was 17 ± 7 months and the average length of follow-up after the 2nd HRCT was 19 ± 9 months. The average total follow-up after diagnosis was 36 ± 14 months. Eighty-four percent of patients received anti-fibrotic therapy, for an average length of 24 ± 23 months.

**Table 1:** Demographic, clinical and radiographic characteristics of the patients at baseline

Variable	Average ± SD	Range
**Age** (years)	71 ± 8	43-89
**Gender** (% male)	72	
**BMI** (kg/m^2^)	30 ± 5	
**Smoking** (pack-years)	22 ± 20	0 - 113
**MDD diagnosis**		
**Clinical-radiographic-pathologic** (%)	37 (30)	
**Clinical-radiographic** (%)	86 (70)	
**FVC** (% pred)	82 ± 21	38 - 132
**TLC** (% pred)	73 ± 16	40 - 118
**DLCO** (% pred)	50 ± 18	17 - 97
**Antifibrotic therapy** (%)	84 (68)	
**Pirfenidone** (%)	39 (32)	
**Nintedanib** (%)	23 (18)	
**Switched therapy** (%)	22 (18)	
**Duration of antifibrotic therapy if any** (months)	24 ± 23	1-82
**Outcomes**		
**Alive without LTx** (%)	69 (56)	
**LTx** (%)	11 (9)	
**Dead** (%)	43 (35)	
**HRCT scan pattern**		
**Definite UIP** (%)	49 (40)	
**Probable UIP** (%)	43 (35)	
**Indeterminate for UIP** (%)	5 (4)	
**Alternative diagnosis** (%)	26 (21)	
**Concomitant emphysema** (%)	46 (37)	

*Abbreviations*: *BMI* body mass index, *DLCO* diffusion capacity, *FVC* forced vital capacity, *HRCT* high resolution CT scan, *LTx* lung transplant, *MDD* multi-disciplinary diagnosis, *TLC* total lung capacity, *UIP* usual interstitial pneumonia

Interobserver agreement between chest radiologists on HRCT fibrosing scoring was good, with a weighted kappa coefficient of 0.64 (S.E. 0.05, C.I. 0.54–0.75).

At the end of the follow-up period, 43 patients (35%) had died, 11 patients (9%) received a LTx and 69 patients (56%) were alive without a LTx. Therefore, the combined incidence of death/LTx was 44%.

**Fig. 1:**
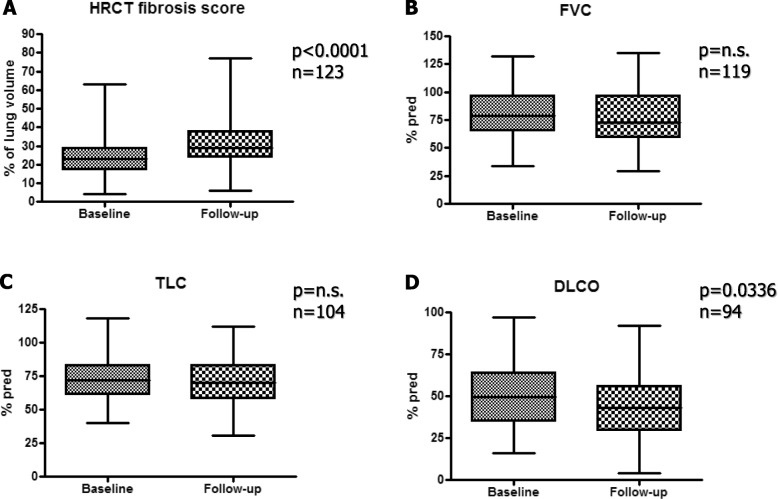
Longitudinal changes from baseline. A. HRCT visual fibrosis score. B. FVC % predicted. C. TLC % predicted. D. DLCO % predicted

Longitudinal radiographic and functional changes were considered (Fig. 1). During the interval between baseline and follow-up HRCT, a significant increase of the HRCT fibrosis score was observed (*p* < 0.0001). During the same interval, only a non-significant decline of FVC and TLC was observed, while the decline of DLCO was significant (*p* = 0.0366).

**Fig. 2:**
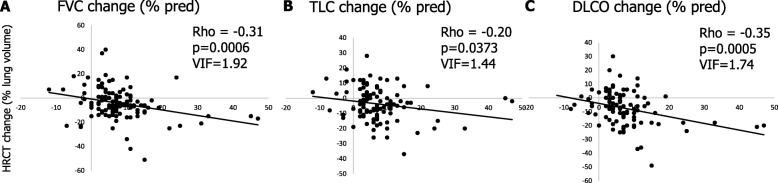
Relationship between HRCT visual fibrosis score longitudinal change and: A. FVC % pred longitudinal change; B: TLC % pred longitudinal change; C: DLCO % pred longitudinal change

The relationships between HRCT changes and lung function changes are shown in Fig. 2. Although these were significant, they were rather weak, ranging between 0.20 and 0.35 of correlation factor. Variance inflation factor (VIF) analysis demonstrated low collinearity between radiographic progression and functional progression: VIF was 1.92 for HRCT fibrosis score vs FVC, 1.74 vs. TLC, and 1.44 vs. DLCO.

We then considered the predictors of survival after the follow-up HRCT (Table 2). Age, gender and BMI were not significant. HRCT fibrosis score a baseline was a significant risk factor towards mortality (*p* = 0.0057). ROC analysis (Table 3) determined that a > 7% increase of HRCT fibrosis score from baseline was the most accurate interval change to predict mortality. This indeed was significant on univariate regression analysis (*p* = 0.0070, Table 2).

**Table 2:** Univariate regression analysis: predictors of lung transplant-free survival after the 2nd HRCT

Variable	Hazard ratio (C.I.)	***p*** value
**Age** (years)	1.01 (0.98–1.05)	n.s.
**Gender** (male)	1.18 (0.65–2.24)	n.s.
**BMI** (kg/m^2^)	0.96 (0.90–1.01)	n.s.
**Baseline HRCT fibrosis score**	1.03 (1.01–1.06)	0.0057
**HRCT fibrosis score change > 7%**	2.10 (1.23–3.62)	0.0070
**Baseline FVC**	0.98 (0.97–0.99)	0.0126
**FVC change ≥ 5%**	2.13 (1.20–3.92)	0.0091
**Baseline TLC**	0.97 (0.95–0.99)	0.0081
**TLC change ≥ 5%**	0.99 (0.96–1.02)	n.s.
**Baseline DLCO**	0.94 (0.94–0.98)	< 0.0001
**DLCO change ≥ 5%**	1.31 (0.66–2.78)	n.s.

**Table 3:** Receive operating characteristics analysis. Predictors of lung transplant-free survival after the 2nd HRCT

Variable	Area under the curve	Sensitivity (C.I.)	Specificity (C.I.)	***p*** value
**HRCT fibrosis score change (> 7%)** **from baseline**	0.61	52% (38–66)	71% (59–81)	0.0266
**FVC change (≥5%)** **from baseline**	0.62	65% (50–78)	60% (47–71)	0.0193
**TLC change (≥5%)** **from baseline**	0.56	55% (39–70)	56% (43–69)	n.s.
**DLCO change (≥5%)** **from baseline**	0.57	70% (53–84)	44% (31–58)	n.s.

Baseline FVC, TLC and DLCO were significantly protective towards mortality at baseline, but only a **≥** 5% longitudinal decline of FVC (*p* = 0.0091) or DLCO (p = < 0.0001) predicted mortality significantly.

In terms of accuracy of mortality prediction, ROC analysis (Table 4) demonstrated that a > 7% longitudinal increase of HRCT fibrosis score was the most specific parameter (71%), while a **≥** 5% decline of DLCO was the most sensitive (70%). However, only HRCT fibrosis score > 7% and FVC decline **≥**5% were actually significant. Areas under the curve for HRCT fibrosis score, FVC, TLC and DLCO longitudinal changes ranged between 0.56 and 0.62 (Table 4).

**Table 4:** Multivariate regression analysis: independent, significant predictors of lung transplant-free survival after the 2nd HRCT

Variable	Hazard ratio (C.I.)	p value
**FVC change ≥ 5%**	2.07 (1.15–3.87)	0.0152
**HRCT fibrosis score change > 7%**	1.92 (1.09–3.41)	0.0260

Multivariate regression analysis (Table 4) demonstrated that a > 7% increase of HRCT fibrosis score and a **≥** 5% decline of FVC were independently and significantly predictive of mortality (*p* = − 0.0260 and *p* = 0.0152, respectively).

## Discussion

In this study, we first demonstrated that a simple HRCT visual fibrosis score is reproducible among chest radiologists with ILD expertise, and also a reliable and responsive predictor of mortality, when longitudinally assessed in IPF. We then demonstrated that no individual pulmonary test closely reflects clinically significant radiographic progression. Although our data showed that a longitudinal decline of FVC predicts worse survival independently from HRCT longitudinal assessment, they also demonstrated that the prognostic accuracy of these methods is far from being optimal.

With prolonged follow-up (average of 3 years) of patients from 2 different centers, we were able to capture a significant amount of progression of disease, as reflected by the high incidence (44%) of death/LTx following the 2nd HRCT, even in the era of systematic anti-fibrotic therapy. This ensured that the study was adequately powered in terms of endpoint events. Moreover, the population studied was not derived from a clinical trial, where trial-ineligible patients would not be included, limiting the representation of the general population of patients with IPF.

Using a variety of statistical approaches, we ascertained that a > 7% increase of HRCT fibrosis score (% of lung volume) is moderately specific in predicting LTx-free survival in IPF. The very significant, longitudinal increase of the score, observed in the context of high incidence of deaths/LTx, reflects that the score is responsive to progression of disease. Finally, an increase of HRCT fibrosis score was strongly associated with mortality/LTx, which makes it highly clinically significant.

HRCT assessment remains the only validated biomarker in the diagnosis and follow-up management of patients with IPF [33]. Hwang et al. previously demonstrated that progression of honeycombing is specifically associated with worse survival, not surprisingly [23]. However, most studies were limited by short follow-up [11] or were not powered enough, due to a limited number of deaths observed [10].

Walsh et al. extensively analyzed inter-observer agreement in terms of interstitial pattern recognition on HRCT [15]. There are however no previous reports about agreement among radiologists in terms of HRCT quantitative visual fibrosis scoring. With a weight kappa coefficient of 0.64, agreement in this study was reassuringly good. However, such results would need to be confirmed in non-academic centers and among radiologists with less experience. At this point, the gold standard for HRCT pulmonary fibrosis quantitative scoring remains to be established.

The availability of a full set of longitudinal pulmonary function data obtained in concomitance to HRCT allowed us to compare radiographic and functional progression. Only moderate correlation was observed between radiographic progression and functional deterioration. Although this may seem concerning in terms of longitudinal functional assessment, it may also point to a complementary role of pulmonary function tests, when coupled with radiographic assessment. Importantly, VIF calculation demonstrated very low collinearity (< 2 for all PFTs) [32] between radiographic progression and functional decline.

Sverzellati et al. found that CALIPER-based HRCT changes and FVC decline are independently predictive of survival in IPF [17]. We expanded these findings by assessing the accuracy of survival prediction of both radiographic and functional progression. We demonstrated that, while radiographic progression is quite specific (71%) to predict mortality, functional progression is relatively more sensitive (65%). On one side, the results of the HRCT visual fibrosis score are importantly reassuring, as automated methods of fibrosis quantification may not be readily available in the majority of centers. On the other hand, the overall accuracy of either method is far from being optimal, with areas under the curve of only 0.61–0.62. This fundamental finding points to a strong need for better refinement of prognostic methods in patients with IPF.

A usual assumption is that HRCT is more sensitive than functional methods to detect progression of disease in IPF [10]. Our finding of superior sensitivity of the FVC decline, compared to the HRCT fibrosis score, in predicting LTx-free survival is relatively surprising. This result can certainly be interpreted a limitation of a visual score, where difficult to detect active disease may be missed, while its physiologic impact may be captured by pulmonary function tests. Another limitation of the visual score is represented by the challenging distinction of pre-existing emphysema from honeycombing. From this perspective, we expect that new automated models of fibrosis scoring, such as AMFM [10], CALIPER [23, 33] and DTA [21], will improve the sensitivity of HRCT in detecting progression of disease. On the other hand, clinical progression may be also related to other factors, such as the development of associated pulmonary hypertension, malnutrition or muscle deconditioning [34].

Fluctuations of lung function tests have also been observed in individual patients [19]. Other factors influencing pulmonary function test variability are acute exacerbations [35] and lack of compliance to treatment. The prognostic results of DLCO may seem disappointing but, with advanced disease, not all patients with IPF are able to perform it [35]. Additional limitations of DLCO measurement include technical variability, need for adjustment in certain conditions such as anemia, and inconsistency when patients are tested in different laboratories [35].

The major limitation of this study is represented by the use of a visual HRCT fibrosis score, which has well known limitations in terms of interobserver agreement and detection of small fibrotic changes, reflected by the low sensitivity of the HRCT visual score on ROC analysis in this study. On the other hand, new texture mapping softwares may not immediately available to all centers [36], nor a specific automated method of fibrosis scoring has been validated yet.

## Conclusions

The HRCT visual fibrosis score is a simple and relatively reliable tool to predict LTx-free survival in IPF, when longitudinally assessed, and with good interobserver agreement. While no individual pulmonary function tests perfectly reflect clinically-significant progression of disease detected on HRCT, this study confirms that the assessment of longitudinal decline of FVC still provides integrated and independent information, enhancing the sensitivity of prognostic models, with no collinearity found. However, concerningly, the accuracy of both HRCT and FVC remains limited, and better prognostic models in IPF are needed. Future studies need to establish whether this can be achieved by using new automated CT scoring methods, or, alternatively, by using a multi-dimensional approach implementing new components, such as exercise capacity and dyspnea scores, with lung function.

## Data Availability

The dataset analyzed during the current study is not publicly available due to privacy restrictions dictated by the Research Ethics Boards. Selected data may be available from the corresponding author on reasonable request.

## Abbreviations

CTD: Connective tissue disease
DLCO: Diffusion capacity of carbon monoxide
FVC: Forced vital capacity
GERD: Gastroesophageal reflux disease
GGO: Ground glass opacity
HRCT: High resolution CT scan
ILD: Interstitial lung disease
IPF: Idiopathic pulmonary fibrosis
LTx: Lung transplant
6MWT: 6-min walking test
PFT: Pulmonary function test
PH: Pulmonary hypertension
TLC: Total lung capacity
UIP: Usual interstitial pneumonia
